# CD147 and Prostate Cancer: A Systematic Review and Meta-Analysis

**DOI:** 10.1371/journal.pone.0163678

**Published:** 2016-09-29

**Authors:** Fei Peng, Hui Li, Zhaoze Ning, Zhenyu Yang, Hongru Li, Yonggang Wang, Fang Chen, Yi Wu

**Affiliations:** 1 People's Hospital of Hunan Province, Chang Sha, Hu Nan Province, China; 2 Reproductive Department, Xiangya Hospital, Central South University, Changsha, China; 3 Urology of Xiangya Hospital, Central South University, Changsha, China; 4 Xiangya Medical School, Central South University, Changsha, China; Thomas Jefferson University, UNITED STATES

## Abstract

**Background:**

Prostate cancer is one of the most common non-cutaneous malignancies in men. We aimed to systemically evaluate the relationship between the expression of CD147 in tissues and the clinicopathological features of prostate cancer.

**Methods and Findings:**

PubMed (1966–2016), EMBASE (1980–2016), the Cochrane Library (1996–2016), Web of Science (1945–2016), China National Knowledge Infrastructure (1982–2016), and the WanFang databases (1988–2016) were searched. Literature quality assessment was performed with the Newcastle-Ottawa Scale. Meta-analysis was performed by using Review Manager 5.3 and Stata 13.0. A total of 12591 prostate cancer patients from 14 studies were included. The results of the meta-analysis showed that there were significant differences in the positive expression rate in the following comparisons: prostatic cancer tissues vs. normal prostate tissues (odds ratio [OR] = 26.93, 95% confidence interval [CI] 7.95–91.20, P < 0.00001), prostatic cancer tissues vs. benign prostatic hyperplasia tissues (OR = 20.54, 95% CI 8.20–51.44, P < 0.00001), high Gleason score vs. low Gleason score (OR = 2.39, 95% CI 1.33–4.27, P = 0.03), TNM III to IV vs. TNM I to II (OR = 9.95, 95% CI 4.96–19.96, P < 0.00001), low or moderate differentiation vs. high differentiation (OR = 8.12, 95% CI 3.69–17.85, P < 0.00001), lymph node metastasis vs. non-lymph node metastasis (OR = 4.31, 95% CI 1.11–16.71, P = 0.03), and distant metastasis vs. non-distant metastasis (OR = 8.90, 95% CI 3.24–24.42, P < 0.00001).

**Conclusion:**

The CD147 positive expression rate was closely related to the clinical characteristics of prostate cancer, but more research is needed to confirm the findings owing to the results of the subgroups.

## Introduction

The incidence of prostate cancer is the second highest among all malignant tumors in men, and it is the most common cancer in men in developed countries [1]. Although the incidence of prostate cancer in developing countries is lower than in developed countries, it has shown a continuous rapid increase in recent years[2]. Prostate cancer prognosis varies significantly among patients according to clinical stage and pathological grade. Early detection and early treatment can help improve patient prognosis[3].

Currently, we mainly use the detection of serum prostate-specific antigen (PSA) to carry out an assessment for prostate cancer. PSA will increase in most clinically significant cases of prostate cancer. However, we hoped to find a more effective index that has a close relationship with the clinical features of prostate cancer[4]. Hence, we performed this meta-analysis.

With the rapid development of molecular biology and immunology techniques in recent years, tumor markers have played an important role in the differential diagnosis, prognosis evaluation, and follow-up of malignant tumors[5].CD147, also known as extracellular matrix metalloproteinase inducer (EMMPRIN), is a member of the immunoglobulin family that is expressed on the surface of many types of tumor cells[6].CD147 has a high expression level in many malignant tumors and there are significant differences both in the intensity and distribution of CD147 staining between malignant tumors and benign lesions; moreover, CD147 expression is reported to correlate with the clinical prognosis of patients with some malignant tumors[7,8,9,10].

To date, there have been some case-control studies that investigated the expression of CD147 in prostate cancer. They found prostate cancer tissues had a higher positivity rate that is significantly different from prostatic hyperplasia and normal prostate tissues, and their authors concluded that the expression of CD147 is related to TNM stage, aggressiveness, distant metastasis, and prognosis of prostate cancer; however, some studies had different conclusions[11,12,13,14,15]. To provide better evidence of the clinical application of CD147 in prostate cancer patients, this meta-analysis was conducted to assess the correlation between CD147 and prostate cancer.

## Methods and Materials

### Criteria for including studies

1. Published case control study or randomized controlled trial that provides original data about CD147 and prostate cancer with clinical pathological characteristics; 2. All cases had complete clinical and pathological data, without radiotherapy or chemotherapy before sampling; 3. Pathological sections were all studied, and CD147 was detected by immunohistochemical staining; 4. When there was duplicate publication or similar information, the best quality study was retained.

### Criteria for excluding studies

1. Animal experiments; 2. The standard of pathological diagnosis was not clear; 3. CD147 was not detected by immunohistochemical staining; 4. A duplicated report, a review, or a case report.

## Search Strategy

We searched PubMed (1966–2016), EMBASE (1980–2016), the Cochrane Library (1996–2016), Web of Science (1945–2016), China National Knowledge Infrastructure (1982–2016), and the WanFang databases (1988–2016). The trials were restricted to humans, but not by date, language, or publication status. The following combined search term was used: (prostate, Prostatic, Prostat*, Prostatomegaly) AND (CD147, extracellular matrix metalloproteinase inducer, EMMPRIN). We combined the term appropriately with MeSH Terms and used an appropriate adjustment for different databases. Details of the search strategies can be found in S1 File.

## Quality Evaluation

The Newcastle-Ottawa quality assessment scale of case control studies (NOS) [16] was adopted to assess the quality of included studies. It has three categories (selection, comparability, and exposure) and eight items. Two researchers performed the quality assessments separately. In the selection category (adequate definition of the cases, representativeness of the cases, selection of controls, definition of controls) and exposure category (ascertainment of exposure, same method of ascertainment for cases and controls, non-response rate), a quality research item received one star, and a comparable category (comparability of cases and controls on the basis of the design or analysis) could receive at most two stars. The quality assessment values ranged from 0 to 9 stars. Each band indicates the percentage of the included studies that met each of these quality criteria.

## Statistical Analysis

Records retrieved from the initial search were independently scanned by two authors to exclude clearly irrelevant studies. Then, the full text articles were independently reviewed by two authors to see if they met the inclusion criteria, and differences of opinion were resolved by a third author. All of the data were extracted independently by two authors. The corresponding author of each study was contacted to provide information on missing or incomplete data. The software Revman 5.3 and Stata 13.0 were used to analyze the data. Results were expressed as odds ratios (OR) and 95% confidence intervals (95% CI). A fixed-effects model was adopted in the case of no evidence of significant heterogeneity (P > 0.1 and I^2^ < 50%); otherwise, a random-effects model was used. If possible, heterogeneity was explored and subgroup analyses were performed. Subgroup analyses were conducted based on patient age and study area. If heterogeneity could not be explored, we conducted a sensitivity analysis to identify the study with the most heterogeneity.

Sensitive analysis was also performed to evaluate the influences of individual studies on the final effect size. When some studies were omitted or subgroup analyses were performed, if no decreases in heterogeneity were observed, a qualitative systematic review method was used to describe the results. All P values were 2-sided, and P < 0.05 was considered significant. Egger’s test was used to assess publication bias (P < 0.05 was considered statistically significant). If publication bias was confirmed, a trim-and-fill method developed by Duval and Tweedie was implemented to adjust for this bias.[17] Then, we replicated the funnel plot with their ‘‘missing” counterparts around the adjusted summary estimate.

## Literature Search

**Fig 1:**
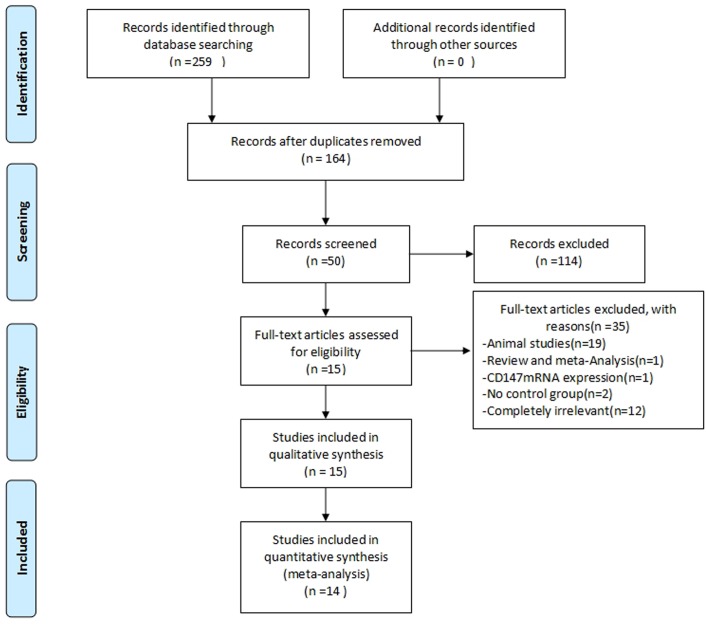
Flow diagram of the literature filtering,14 studies are identified for analysis.

A total of 259 studies were identified, and 95 studies were excluded because of duplication. After reading the titles and abstracts, 114 studies were excluded. Fifty full text studies were carefully reviewed (excluded for being animal studies [n = 19]; reviews and meta-analyses [n = 1]; reported CD147 mRNA expression [n = 1]; no control group [n = 2]; and completely irrelevant [n = 12]). Finally, 15 trials were included for qualitative analysis and 14 trials were included for quantitative analysis (Fig 1).

## Characteristics and Risk of Bias of Included Studies

A total of 15 studies [9,10,11,12,13,14,15,18,19,20,21,22,23,24,25] were identified for qualitative analysis. The study by Bauman et al. was not included in the quantitative analysis because it provided the mean optical density of CD147 and not the positive expression rate of CD147. Therefore, 14 studies [9,10,11,12,13,14,18,19,20,21,22,23,24,25] were included for quantitative analysis, including 12,591 prostate cancer tissues, 298 benign prostatic hyperplasia tissues, and 175 normal prostate tissues. Six articles provided a positivity rate of CD147 expression in prostate cancer and normal prostate tissues, 8 articles reported on CD147 expression in prostate cancer and benign prostatic hyperplasia, 12 articles involved Gleason score, 6 articles involved TNM stage, 5 articles involved differentiation, 6 articles involved lymph node metastasis, and 2 articles involved distant metastasis.

Eleven of the studies were performed in Asia and 3 elsewhere. All 14 studies adopted immunohistochemistry (IHC) as the detection method, but the method for judging negative and positive staining was different among studies. For example, Wang used an IHC score. However, Nelma Pertega-Gomes, Grupp K, and Weide Zhong used cell staining intensity (Nelma Pertega-Gomes and Grupp K’s cut-off value was 0%, while Weide Zhong’s was 5%). The source of antibodies in all included studies was primarily three places (Fourth Military Medical University, Sigma, and ZYMED). The tissue slice thickness across the studies had some minor differences.

We used the NOS scale to evaluate the literature, and all of the studies had a score greater than 5, indicating that the quality of the literature is high. The statement “High CD147 expression” indicated that the positive rate of CD147 expression was high in the population and “Low CD147 expression” indicated that the positive rate of CD147 expression was low in the population.

We performed a subgroup analysis according to three aspects: country, antibody source, and positivity scoring system because these three aspects may affect the results. The characteristics of the studies are presented in Table 1 and the NOS results can be seen in Table 2.

**Table 1:** Characteristics of included studies.

First Author	Year	Country	Prostate cancer (age)	benign prostatic hyperplasia (age)	Control group (age)	Test Methor (slice thickness)	Criterion of judgment for CD147 expressing	NOS score	Indicators reported	Antibody source
Weide Zhong[18]	2009	China	101(73.5)	90(unclear)	36(unclear)	IHC(unclear)	negative:0~1 points(IHC Score*)	8	①②③④⑤	Fourth Military Medical University
Zhen Ma[19]	2014	China	60(58–81)	30(55–82)	-	IHC(4 um)	negative:Ratios of stained cell nunber≤5%	7	②③⑥	ZYMED
Q wang[12]	2015	China	54(58.1±3.9)	40(57.8±4.3)	20(56.9±5.11)	IHC(unclear)	negative:0~1 points(IHC Score*)	8	②④⑤	Baygene Biotech
Zhaodong Han[11]	2009	China	62(73.9±12.1)	30(unclear)	-	IHC(3 um)	negative:0 points(IHC Score*)	7	②③④⑤	Fourth Military Medical University
Michele C Madigan[9]	2008	Australia	120(46–76)	15(53–72)	20(62–84)	IHC(5 um)	negative:Ratios of stained cell nunber≤25%	7	①②③	ZYMED
Nelma Pertega-Gomes[10]	2011	Portugal	171(46–74)	-	14(unclear)	IHC(4 um)	negative:0% of immunoreactive cells	7	③	ZYMED
Ming-en Feng[20]	2010	China	60(59–89)	15(59–80)	-	IHC(4 um)	unclear	7	②③⑥	ZSGB-BIC,Beijing
Junbo Huang[21]	2015	China	56(58.6±4.2)	56(56.9±5.4)	-	IHC(5 um)	negative:<3 points(IHC Score*)	8	②③④⑤⑥	Sigma
Weide Zhong[13]	2012	China	240(61.9)	-	20(unclear)	IHC(5 um)	negative:<5% of immunoreactive cells	6	①③⑦	Fourth Military Medical University
Chongyue Cai[22]	2015	China	56(67.5±4.78)	22(67.7±6.50)	-	IHC(5 um)	negative:1~3 points(IHC Score*)	6	①③⑥⑦	BOSTER,Wuhan
Zhong Chen[23]	2013	China	97(68.6)	-	45(unclear)	IHC(4 um)	negative:0~1 points(IHC Score*)	7	①④	Sigma
Jun Zou[24]	2007	China	62(74)	15(unclear)	-	IHC(unclear)	negative:0~1 points(IHC Score*)	6	②③④⑤	Fourth Military Medical University
Xuecheng Bi[14]	2011	China	300(66.2)	-	20(65.3)	IHC(3 um)	negative: ≤5%of immunoreactive cells	7	①③⑥	Fourth Military Medical University
Grupp K[25]	2013	Germany	11152(53.4)	-	-	IHC(unclear)	negative:scores had absence of CD147 staining	8	③⑥	Dako
Tyler M.Bauman[15]	2013	USA	190	48	96	IHC(unclear)	-	8	③④⑥	Meridian Life Science, Memphis

Notes: IHC = immunohistochemistry; IHC Score* = A*B (positively stained CD147, mainly located in the plasma membrane and cytoplasm, exhibiting a yellowish or brown color. Under a high magnification field, each slide of the specimen was examined individually by three pathologists and graded according to varying staining states in the plasma membrane and the cytoplasm. Unstained, weakly stained, moderately stained, and deeply stained specimens were given 0, 1, 2, and 3 points, respectively, designated as score A. Ratios of stained cell numbers to unstained cell numbers: when ≤ 5%, between 6%–25%, between 26%–50%, or > 51% received 0, 1, 2, or 3 points, respectively, designated as score B. The final score is the product of A multiplied by B); ①. CD147 with prostatic cancer tissues and normal prostate tissues; ②. CD147 with prostatic cancer tissues and benign prostatic hyperplasia tissues; ③. CD147 with Gleason score of prostatic cancer tissues; ④. CD147 with TNM stage of prostatic cancer tissues; ⑤. CD147 with differentiation of prostatic cancer tissues; ⑥. CD147 with lymph node metastasis of prostatic cancer tissues; ⑦. CD147 with distant metastasis of prostatic cancer tissues.

**Table 2:** NOS score of included studies.

Column	Entries	First author														
		①	②	③	④	⑤	⑥	⑦	⑧	⑨	⑩	⑪	⑫	⑬	⑭	⑮
	Is the definition adequate	☆	☆	☆	☆	☆	☆	☆	☆	☆	☆	☆	☆	☆	☆	☆
	Representativeness of the cases	☆	☆	☆	☆	☆	☆	☆	☆	☆	☆	☆	☆	☆	☆	☆
Section	Selection of controls	☆				☆	☆			☆	☆	☆		☆		
	Definition of controls	☆	☆	☆	☆	☆	☆	☆	☆	☆	☆	☆	☆	☆	☆	☆
Comparability	Comparability of cases and controls on the basis of the design and analysis	☆	☆	☆	☆	☆	☆	☆	☆	☆	☆	☆	☆	☆	☆☆	☆☆
	Ascertainment of exposure	☆	☆	☆	☆	☆	☆		☆	☆	☆		☆	☆	☆	☆
Exposure	Same method of ascertainment for cases and controls	☆	☆	☆	☆	☆	☆	☆	☆	☆	☆	☆	☆	☆	☆	☆
	Non-Response rate	☆	☆	☆	☆	☆	☆	☆	☆	☆	☆	☆	☆	☆	☆	☆
Total scores		8	7	7	7	8	8	6	7	8	8	7	7	8	8	8

Notes: ①. Weide Zhong 2009; ②. Zhen Ma 2014; ③. Qwang 2015; ④. Zhaodong Han 2008; ⑤. Michele C. Madigan 2008; ⑥. Nelma Pertega-Gomes 2011; ⑦. Ming-en Feng 2010; ⑧. Junbo Huang 2015; ⑨. Weide Zhong 2011; ⑩. Chongyue Cai 2015; ⑪. Zhong Chen 2013; ⑫. Jun Zou 2007; ⑬. Xuecheng Bi 2011; ⑭. Grupp K 2013;⑮Tyler M.Bauman 2015

### CD147 in prostate cancer and normal prostate tissues

**Fig 2:**
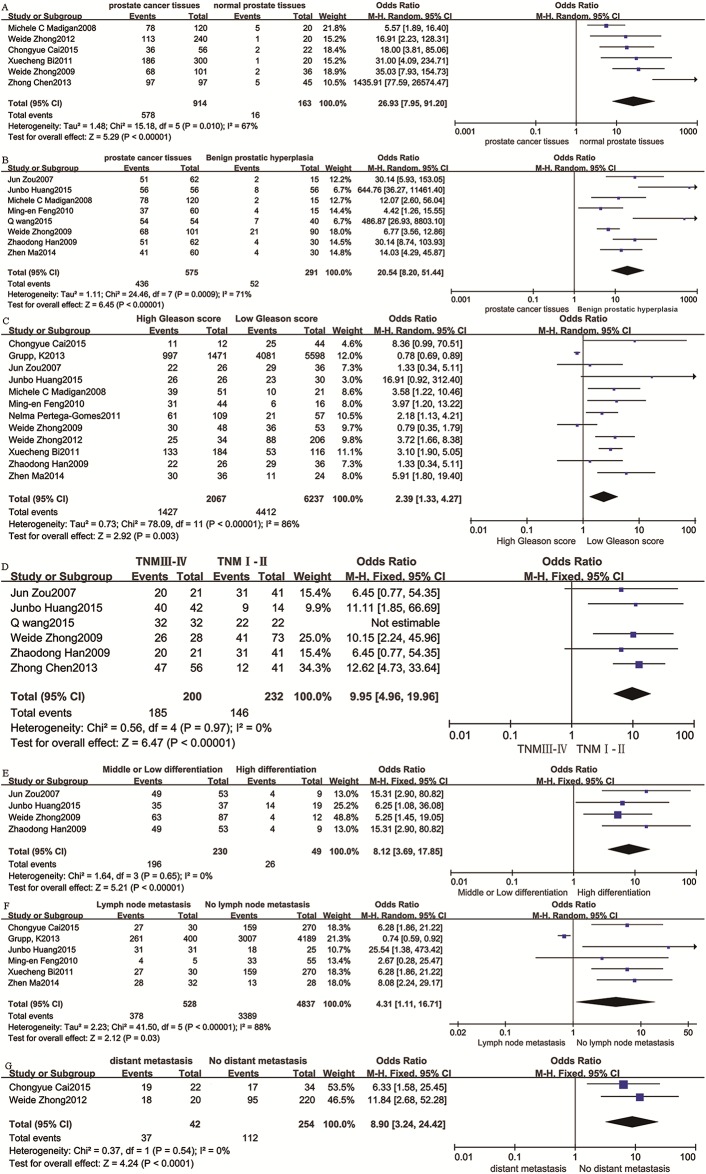
Forest plot of OR was assessed for association between CD147 and clinical pathologic features. **A** Forest of CD147 expression in prostatic cancer tissues vs normal prostate tissues.CD147 expression was higher in prostatic cancer than normal prostate tissues(OR = 26.93,95%CI = (7.95,91.20),P<0.00001).**B** Forest of CD147 expression in prostatic cancer tissues vs benign prostatic hyperpiasia tissues.CD147 expression was higher in prostatic cancer than benign prostatic hyperpiasia tissues(OR = 20.54,95% = (8.20,51.44),P<0.00001).**C** Forest of CD147 expression and Gleason score.CD147 expression was associated with Gleason score of prostatic cancer(OR = 2.39,95%CI = (1.33,4.27),P = 0.03).**D** Forest of CD147 expression and TNM stage.CD147 expression was associated with TNM stage of prostatic cancer(OR = 9.95,95%CI = (4.96,19.96),P<0.00001).**E** Forest of CD147 expression and tumor differentiation.CD147 expression was associated with tumor differentiation of prostatic cancer(OR = 8.12,95%VI = (3,69,17.85),P<0.00001).**F** Forest of CD147 expression and lymph node metastasis.CD147 expression was associated with lymph node metaatasis of prostatic cancer(OR = 4.31,95%CI = (1.11,16.71),P = 0.03).**G** Forest of CD147 expression and diatal metastasis.CD147 expression was associated with diatal metastasis of prostatic cancer(OR = 8.90,95%CI = (3.24,24.42),P<0.00001).

Six studies [9,13,14,18,22,23] reported the positivity rate of CD147 in prostate cancer tissues and normal prostate tissues, including 914 prostate cancer tissues and 163 normal prostate tissues. There was significant heterogeneity (P = 0.01, I^2^ = 67%), and a random-effects model showed the CD147 positive expression rate in prostate cancer tissues was higher than that in normal prostate tissues (OR = 26.93, 95% CI = 7.95–91.20, P < 0.00001) (Fig 2A). To further confirm the stability of the result, a sensitivity analysis was performed to evaluate the influences of individual studies on the final effect. The sensitivity analysis showed that, irrespective of which study was removed, the result was almost the same (S1 Fig), indicating that the result was stable. In addition, subgroup analysis found the CD147 positive expression rate was significantly different between prostate cancer tissues and normal prostate tissues in all groups (S1 Table). Overall, the CD147 positive expression rate in prostate cancer tissues was higher than that in normal prostate tissues.

### CD147 with prostate cancer and benign prostatic hyperplasia tissues

Eight trials [9,11,12,18,19,20,21,24] reported the difference of CD147 positivity rate between prostate cancer and benign prostatic hyperplasia, including 575 prostate cancer tissues and 291 benign prostatic hyperplasia tissues. A random-effects model showed the CD147 positive expression rate in prostate cancer tissues was higher than that in benign prostatic hyperplasia tissues (OR = 20.54, 95% CI = 8.20–51.44, P < 0.00001) with significant heterogeneity (P = 0.0009, I^2^ = 71%) (Fig 2B). A sensitivity analysis was performed to evaluate the influences of individual studies on the final effect, which showed that irrespective of which study was ruled out, the result was almost the same (S2 Fig), indicating that the result was stable. Subgroup analysis showed that the CD147 positive expression rate was significantly different between prostate cancer tissues and benign prostatic hyperplasia tissues except in the group of other antibodies (S1 Table). Therefore, more research is needed to confirm whether the CD147 positive expression rate is higher in prostate cancer tissues than in benign prostatic hyperplasia tissues.

### CD147 with the Gleason score of prostate cancer tissues

The Gleason score is an important reference index for the treatment of patients with prostate cancer, and it is highly correlated with biological behavior and prognosis[24]. The high Gleason score group consists of those with a score > 7 and 3 + 4, and the low Gleason score group contains those scoring < 7 and 4 + 3. Twelve studies [9,10,11,13,14,18,19,20,21,22,24,25], including 8,304 prostate cancer tissues, were included in the analysis. There was significant heterogeneity (I^2^ = 86%, P < 0.00001), and a random-effects model was used. The high Gleason score group had a higher positive rate of CD147 expression (OR = 2.39, 95% CI = 1.33–4.27, P = 0.003) (Fig 2C). A sensitivity analysis showed that the result was stable (S3 Fig). Subgroup analysis showed a significant difference between high and low Gleason score in the groups of Asian, Fourth Military Medical University, ZYMED, and ratios of stained cells, but there was no significant difference in the groups of non-Asian, Sigma, other antibodies, or IHC score (S1 Table). Thus, the conclusion that CD147 positive expression rate is higher in prostate cancer with a high Gleason score than in cases with a low Gleason score should be interpreted cautiously.

### CD147 with TNM stage of prostate cancer tissues

TNM stage is an international standard for tumor staging that can be used to select the best treatment for prostate cancer. The tumor T stage describes the primary tumor lesions[26]. Prostate cancer with TNM T III and IV stage has a significantly poorer prognosis than TNM stage I and II[27,28]. Six studies [11,12,18,21,23,24] including 432 prostate cancer patients as shown in (Fig 2D) found there was a significant difference in the positive rate of CD147 expression between the two T stage groups (OR = 9.95, 95% CI 4.96–19.96, P < 0.00001) without heterogeneity (I^2^ = 0%, P = 0.97). Sensitivity analysis found the result was stable (S4 Fig). In addition, subgroup analysis showed that there is a statistically significant difference between TNM T III and IV stage in all groups (S1 Table). Overall, the CD147 positive expression rate in TNM III and IV is higher than in TNM I and II.

### CD147 with differentiation of prostate cancer tissues

Differentiation is used to describe the degree of malignancy of the tumor. High differentiation indicates that the degree of differentiation of the tumor cells is close to that of normal cells with a low degree of malignancy. Low differentiation indicates that the tumor is highly malignant[29]. Five studies[11,12,18,21,24] were included in this analysis, with 333 prostate cancer tissues (Fig 2E). The CD147 positive expression rate in tumors with moderate or low differentiation was higher than in those with high differentiation (OR = 8.12, 95% CI 3.69–17.05, P < 0.00001) without heterogeneity. Sensitivity analysis showed the result was stable (S5 Fig). In addition, subgroup analysis showed that there was a significant difference between high differentiation and low differentiation in all groups (S1 Table). Overall, the CD147 positive expression rate in tumors with low differentiation was higher than in those with high differentiation.

### CD147 with lymph node metastasis of prostate cancer tissues

Prognosis is often not good when patients with prostate cancer have lymph node metastasis, which is why it is very important to diagnose the condition during the early stages[30]. Six studies [13,14,19,20,21,25] were included in this analysis, with 5,365 prostate cancer tissues (as shown in Fig 2F). There was significant heterogeneity (P < 0.00001, I^2^ = 88%), and a random-effects model showed that the CD147 positive expression rate in prostate cancer tissues with lymph node metastases was higher than that in normal prostate tissues (OR = 4.31, 95% CI 1.11–16.71, P = 0.03). Sensitivity analysis showed that the result was stable (S6 Fig). Subgroup analysis found there was not a significant difference between lymph node metastasis and non-lymph node metastasis in the groups of other antibodies and ratios of stained cells (S1 Table). Therefore, the conclusion that the CD147 positive expression rate in prostate cancer with lymph node metastasis is higher than that in cases with non-lymph node metastasis should be interpreted cautiously until it is confirmed.

### CD147 with distant metastasis of prostate cancer tissues

Three methods of distant metastasis of prostate cancer are direct spread, blood metastasis, and lymph node metastasis. Bone metastases are the most likely to occur[31]. Only two studies [13,22] reported this outcome in a total of 296 prostate cancer tissues. As shown in (Fig 2G), there was a significant difference in the positive rate of CD147 expression between the two groups (OR = 8.90, 95% CI 3.24–24.42, P < 0.00001) without heterogeneity (I^2^ = 0%, P = 0.54). Sensitivity analysis showed that the result was stable (S7 Fig). In addition, subgroup analysis showed that there was a significant difference between cases with distant metastases and those without distant metastases in all groups (S1 Table). Overall, the CD147 positive expression rate in prostate cancer with distant metastases was higher than that in cancers without distant metastases.

## Subgroup Results

In subgroup analysis, we analyzed the studies according to country, antibody source, and IHC scoring system(S1 Table). For the country subgroup analysis, only the results of the Gleason score model may be changed because of the area the study was conducted in (in Asia, OR = 3.57, 95% CI [2.52–5.06], P < 0.00001; in non-Asia, OR = 1.66, 95% CI [0.63–4.35], P = 0.3). In the subgroup analysis by antibody source, the conclusions for prostatic cancer tissues vs. benign prostatic hyperplasia tissues, high Gleason score vs. low Gleason score, and lymph node metastasis vs. non-lymph node metastasis may change when different antibodies are used. In the subgroup analysis by scoring system subgroup, we found that there was no significant difference between high Gleason score and low Gleason score for CD147 positive expression rate (OR = 1.59, 95% CI [0.93–2.73], P = 0.09). Furthermore, no significant difference was found in the group of ratios of stained cells between lymph node metastasis and non-lymph node metastasis (OR = 2.98, 95% CI [0.63–14.07], P = 0.17).

## Sensitivity Analyses

Influence analyses, in which one study is removed at a time, were performed for each meta-analysis to evaluate the stability of the results. These analyses showed that the corresponding OR was not significantly altered and suggested that our results are stable.

## Publication Bias

Egger’s test (as shown in Table 3) and funnel plots were used to evaluate the publication bias of these studies. Publication bias was found in the Gleason score (P = 0.003) and TNM stage (P = 0.038) but the other outcomes suggested no evidence of publication bias. We used a trim-and-fill method to adjust for the publication bias. For the Gleason score, we first excluded four studies that had a smaller number of patients. The adjusted summary showed there was no significant difference between high and low Gleason score (OR = −0.083, 95% CI −0.197 to 0.032, P = 0.156) and there was no heterogeneity. Then, we added four studies that showed the opposite conclusion. The adjusted summary suggested there was a significant difference between cancers with a high and a low Gleason score (OR = −0.237, 95% CI −0.346 to −0.129, P < 0.0001) without heterogeneity. However, our conclusion about the Gleason score should be interpreted with caution and it needs more research to support it. The adjusted results of the TNM stage, after one article was excluded and nothing was added, suggested a similar conclusion, that there was a significant difference between TNM III and IV and TNM I and II. This was consistent with our prior conclusion and indicates our results are statistically robust.

**Table 3:** Egger’s test of funnel plot asymmetry.

Clincopathological parameters	P value
Pca tissues vs normal prostate tissues	0.143
Pca tissues vs Bph tissues	0.208
Gleason score	0.003
TNM stage	0.038
Differentiation	0.365
Lymph node metastasis	—
distant metastasis	—

## Discussion

Prostate cancer is the most common malignancy in men and a major cause of cancer deaths.[32]. Moreover, there is no effective therapy for prostate cancer with metastasis.[33] CD147, a new marker receiving attention in current research on tumors, is highly expressed in malignant tumors.[34] It can promote the invasion and metastasis of tumors by promoting the expression of MMPs.[35] Moreover, it plays an important role in protecting tumor cells that are involved in glycolysis.[36] Recent studies have found that CD147 is closely associated with multiple cancers, including gastric cancer, hepatocellular carcinoma, and ovarian cancer.[37–39]

Our study is a meta-analysis and systematic review that studied the relationship between CD147 and prostate cancer. The results suggest that CD147 expression is statistically significantly different in prostatic cancer tissues vs. normal prostate tissues, in TNM III to IV vs. TNM I to II, low or moderate differentiation vs. high differentiation, and distant metastasis vs. non-distant metastasis. Subgroup analysis resulted in the same conclusions. As for other indicators, the conclusions should be interpreted cautiously because some subgroup analyses had the opposite results.

However, the question as to whether CD147 expression is higher in prostate cancer tissues remains inconclusive. Although our study demonstrated CD147 expression is high in prostate cancer tissues, the results of other trials are not consistent with our results. For example, the study of Grupp et al.[25] indicated that decreased CD147 expression is linked to ERG-fusion positive prostate cancer. In addition, Pertege-Gomes et al.[10] and Bauman et al.[15] have shown that CD147 expression in normal prostate tissue is not only higher than that in benign prostatic hyperplasia tissues but also higher than that in prostate cancer tissues, and this conclusion is contrary to the conclusion we reached. This area needs more research to clarify the issue.

Moreover, Bauman et al.[15] reported that decreased expression of CD147 in prostate cancer tissues is related to advanced pathologic stage and higher Gleason score. Furthermore, the decreased content of CD147 in prostate cancer tissues was confirmed.

Plasma membrane-localized CD147 was quantitatively detected in the study by Bauman et al. However, CD147 was thought to be positively expressed in other trials as long as it was detected and this difference in approaches may lead to a different conclusion. In this study, we investigated the positive rate of CD147 expression. This meant that as long as CD147 was detected in the sample, we regarded the sample as a “positive” one. In the positive samples of prostate cancer tissues, they may have had a low content of CD147 that was quantitatively detected. Conversely, the positive samples of normal prostate tissues and benign prostatic hyperplasia tissues may have had a higher content of CD147, although the positive rate of CD147 expression was lower. Therefore, our conclusions are not in conflict with the study of Bauman et al.

For the Gleason score, we found the expression of CD147 was higher in high Gleason score prostate cancer than in those with a low Gleason score[40]. However, there was publication bias in the studies reporting expression and Gleason score. We also found some studies classified “Gleason score = 7” as a low Gleason score, while others considered it to be a high Gleason score. This may be the reason for the publication bias. In addition, analyses of different subgroups had different conclusions. There was not a statistical difference between high Gleason score and low Gleason score in the groups of non-Asian, Sigma, other antibodies, or IHC score. Therefore, our conclusion about the relationship between Gleason score and CD147 should be interpreted with caution.

For lymph node metastasis, our conclusion changes when the article by Grupp et al. was included. When the study by Grupp et al. was included in our analysis, the random-effects model shows that CD147 expression was associated with lymph node metastasis of prostate cancer, and the fixed-effects model showed that they are not independent. After the exclusion of the study by Grupp et al., the random-effects model and the fixed-effects model both show that they were related; hence, more research is needed to confirm this finding.

Other outcomes, such as TNM stage, tissue differentiation, and distant metastasis, all have a correlation with CD147 expression regardless of whether a random-effects or a fixed-effects model was used. Overall, four outcomes had heterogeneity (prostate cancer vs. normal prostate, prostate cancer vs. benign prostatic hyperplasia, high Gleason score vs. low Gleason score, lymph node metastasis vs. no lymph node metastasis); therefore, we used random-effects models for analysis.

In addition, Dogru et al.[41] found that serum CD147 levels were significantly higher in patients with prostate cancer compared to healthy individuals by using ELISA, but there was no significant difference between men with prostate cancer and those without considering urinary CD147 levels.

In addition to measuring CD147 in tissues by IHC, some studies examined mRNA expression of *CD147* using reverse transcriptase-polymerase chain reaction methods. Cai et al.[22] studied the expression of *CD147* mRNA in prostate cancer and found that the expression of *CD147* mRNA was closely related to TNM stage and tumor differentiation in prostate cancer patients. In addition, the relationship between preoperative PSA and prostate cancer was mentioned in other studies but it remains to be confirmed whether there is an association between them.

Another meta-analysis[42] indicates that CD147 is a tissue biomarker for the prognosis of prostate cancer that can predict the outcome of patients. They found CD147 expression to be correlated with all-cause mortality (HR, 2.63; 95% CI, 1.19–5.81) and disease-free survival (HR, 5.84; 95% CI, 3.41–9.99), which shows that CD147 expression has a close association with prostate cancer. To a certain extent, this is consistent with our conclusion that indicates the prognosis of prostate cancer may be worse when CD147 is expressed. Overall, CD147 positive rate is highly correlated with clinical and pathological features of prostate cancer.

Our study is a meta-analysis about the relationship between CD147 and prostate cancer. A total of 14 trials were included. The quality of all of the trials is high, with a NOS above 5 points. Moreover, all included studies used an immunohistochemical method. Because IHC is only a partially quantitative method, we did not perform an analysis for quantitative data. However, a description and explanation was performed for relevant quantitative studies.

There are some limitations of our study. First, 12,591 prostate cancers were included in the 14 studies, but not every outcome had that many samples. For some outcomes (such as lymph node metastasis), there were a relatively small number of samples, and therefore, we need more trials to confirm our conclusions. Second, although we tried to collect all relevant data from these studies, some data could still be missing, indicating possible, unavoidable publication bias. Third, the cut-off value was different among some studies, which had inconsistent definitions for “negative” and “positive,” leading to between-study heterogeneity. Therefore, the results need to be interpreted with caution. In addition, some unpublished trials that had negative outcomes were not included.

## Conclusion

The results of this review suggest that CD147 positivity is higher in prostate cancer than in benign prostatic hyperplasia and normal prostate tissue. Furthermore, the positivity rate of CD147 expression is associated with Gleason score, TNM stage, differentiation, lymph node metastasis, and distant metastasis. However, the conclusion that CD147 positivity rate was higher in prostatic cancer tissues vs. benign prostatic hyperplasia tissues, high Gleason score vs. low Gleason score, and lymph node metastasis vs. non-lymph node metastasis should be interpreted with caution and needs to be confirmed owing to the different results in the subgroup analyses.

## Supporting Information

S1 Fig Sensitivity analysis of CD147 with prostate cancer and normal prostate tissues. (TIF)

S2 Fig Sensitivity analysis of CD147 with prostate cancer and Benign prostatic hyperplasia tissues. (TIF)

S3 Fig Sensitivity analysis of CD147 with Gleason score of prostate cancer tissues. (TIF)

S4 Fig Sensitivity analysis of CD147 with TNM stage of prostate cancer tissues. (TIF)

S5 Fig Sensitivity analysis of CD147 with differentiation of proatate cancer tissues. (TIF)

S6 Fig Sensitivity analysis of CD147 with lymph node metastasis of prostate cancer tissues. (TIF)

S7 Fig Sensitivity analysis of CD147 with distant metastasis of prostate cancer tissues. (TIF)

S1 File Search terms and the number of studies identified from (A) Pubmed (B) EMBASE (C) Cochrane Library(D)Web Of Science(E)China National Knowledge Infrastructure(F)The WanFang Databases. (DOCX)

S2 File PRISMA checklist. (DOC)

S1 Table The results of subgroup analysis. (DOCX)
