# Supplementary figures and images for: CD147 and Prostate Cancer: A Systematic Review and Meta-Analysis
Source: PLoS One. 2016 Sep 29;11(9):e0163678. doi: 10.1371/journal.pone.0163678 (PMC5042541; doi:10.1371/journal.pone.0163678)

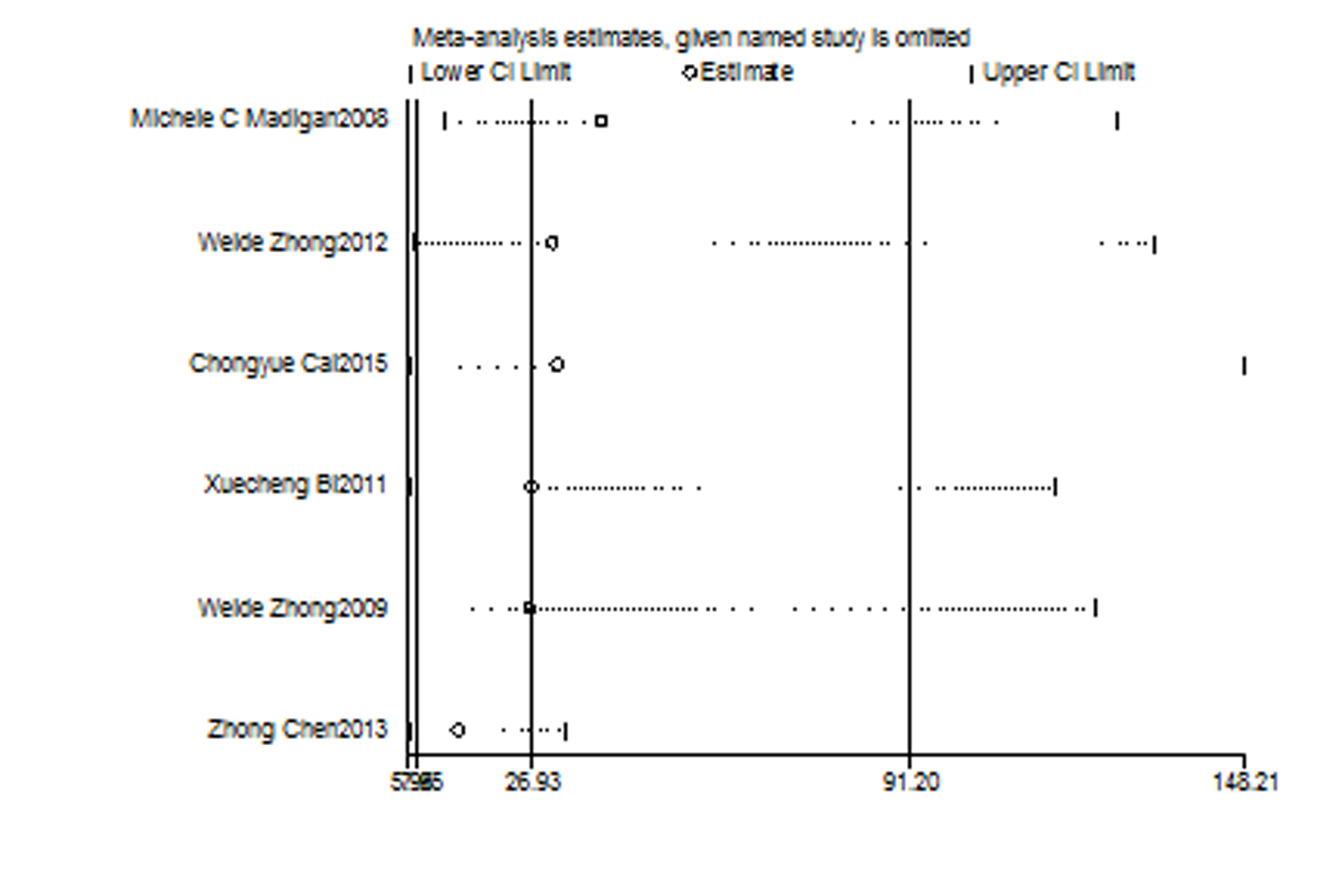

Supplement: S1 Fig — (TIF) [file pone.0163678.s001.tif]

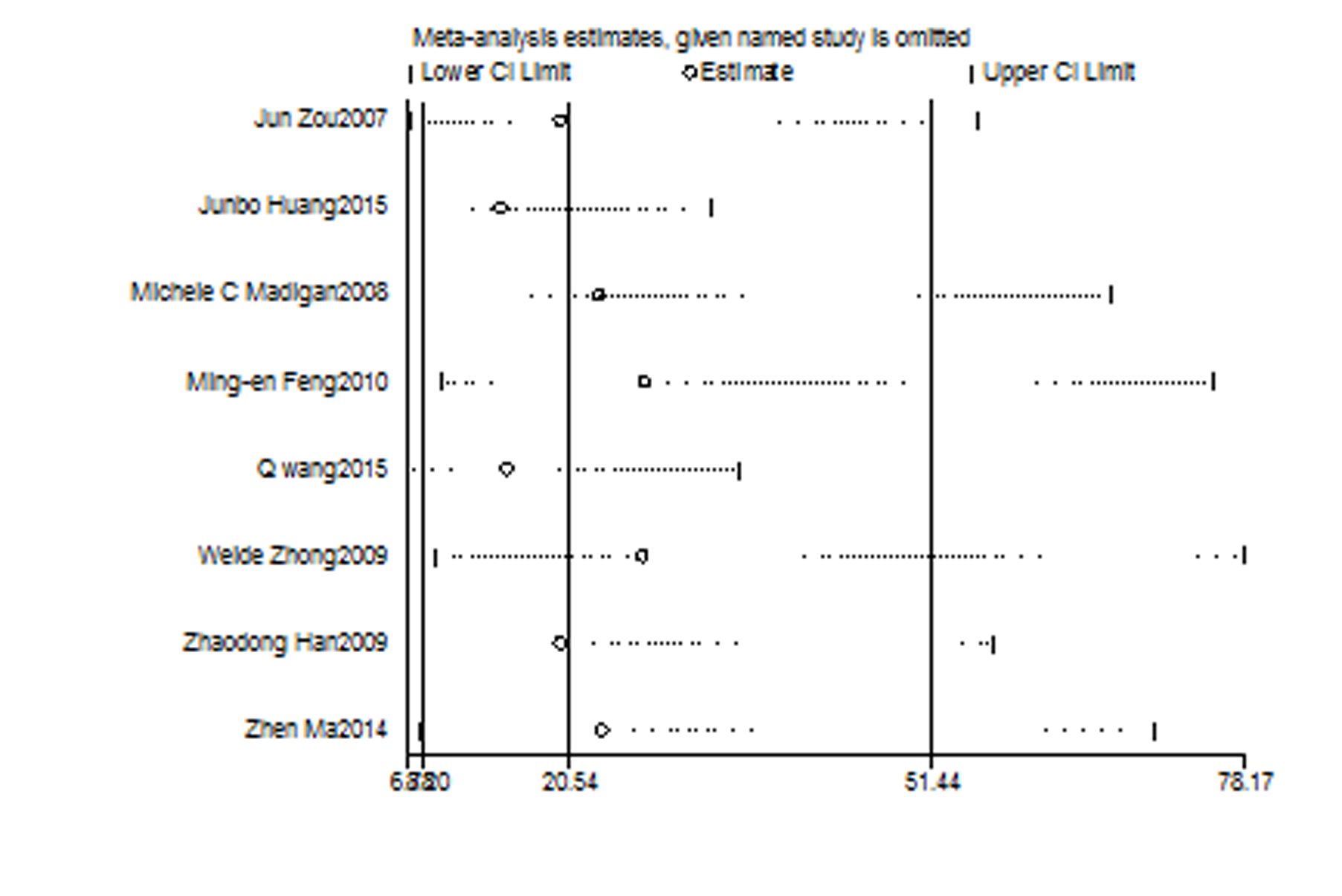

Supplement: S2 Fig — (TIF) [file pone.0163678.s002.tif]

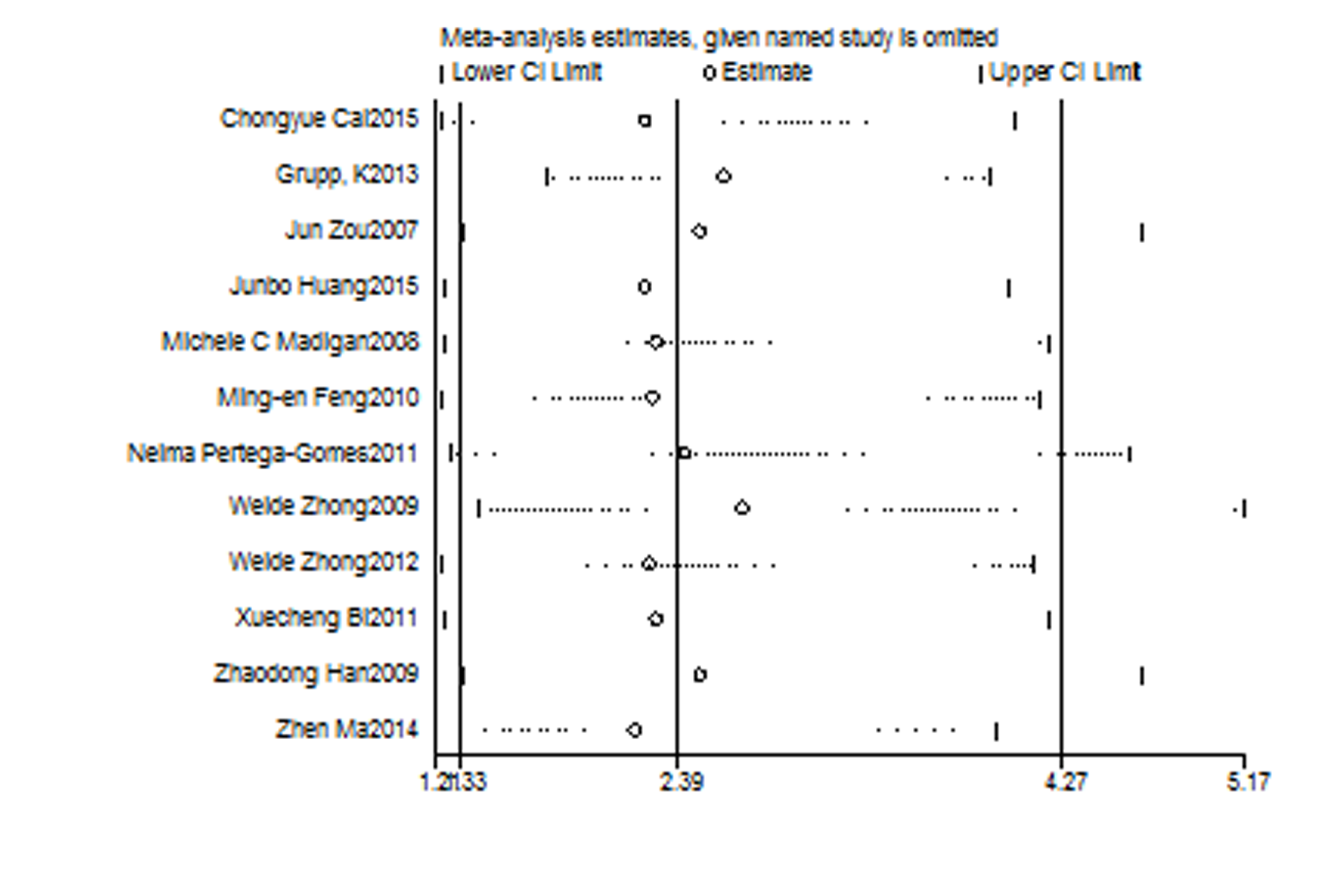

Supplement: S3 Fig — (TIF) [file pone.0163678.s003.tif]

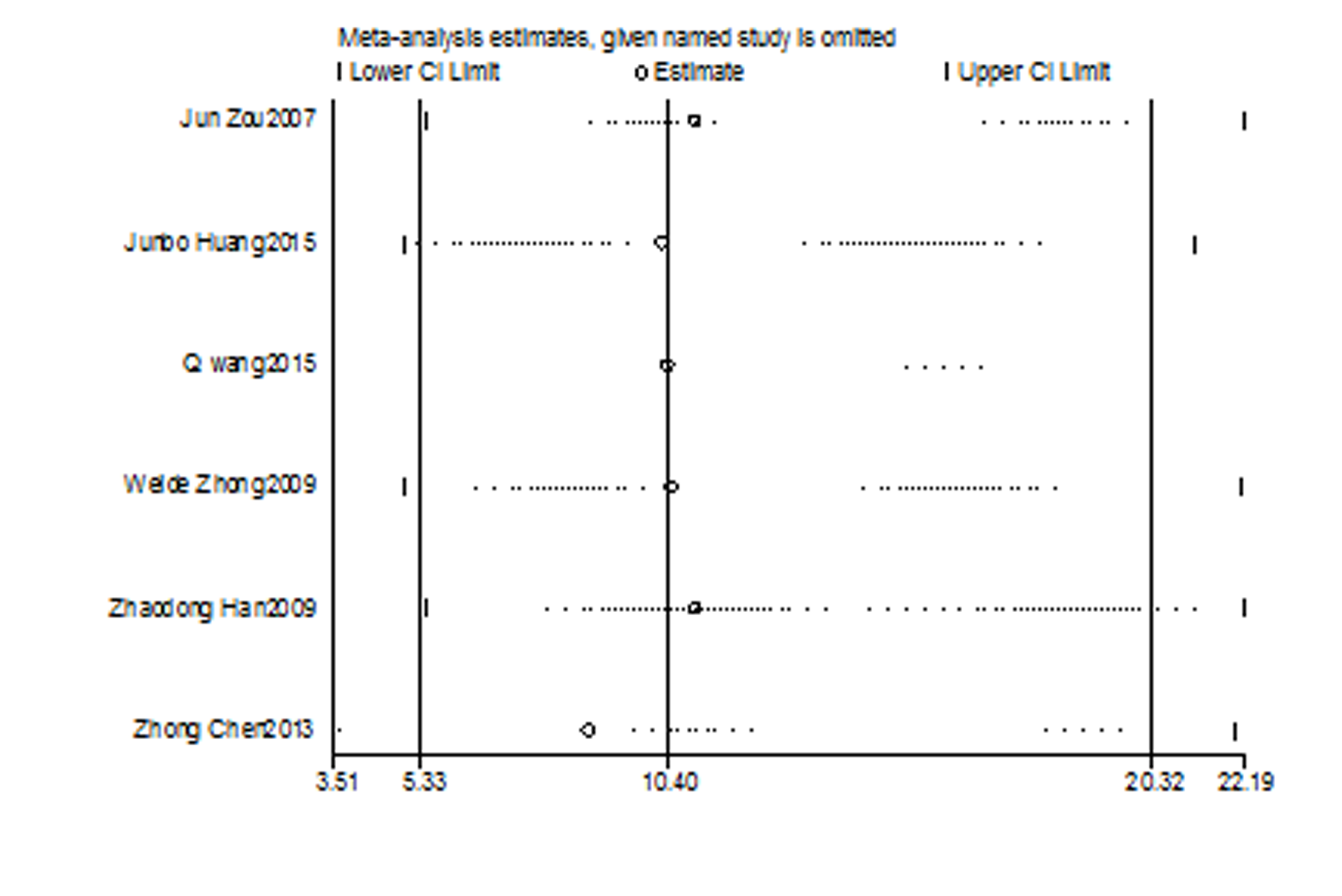

Supplement: S4 Fig — (TIF) [file pone.0163678.s004.tif]

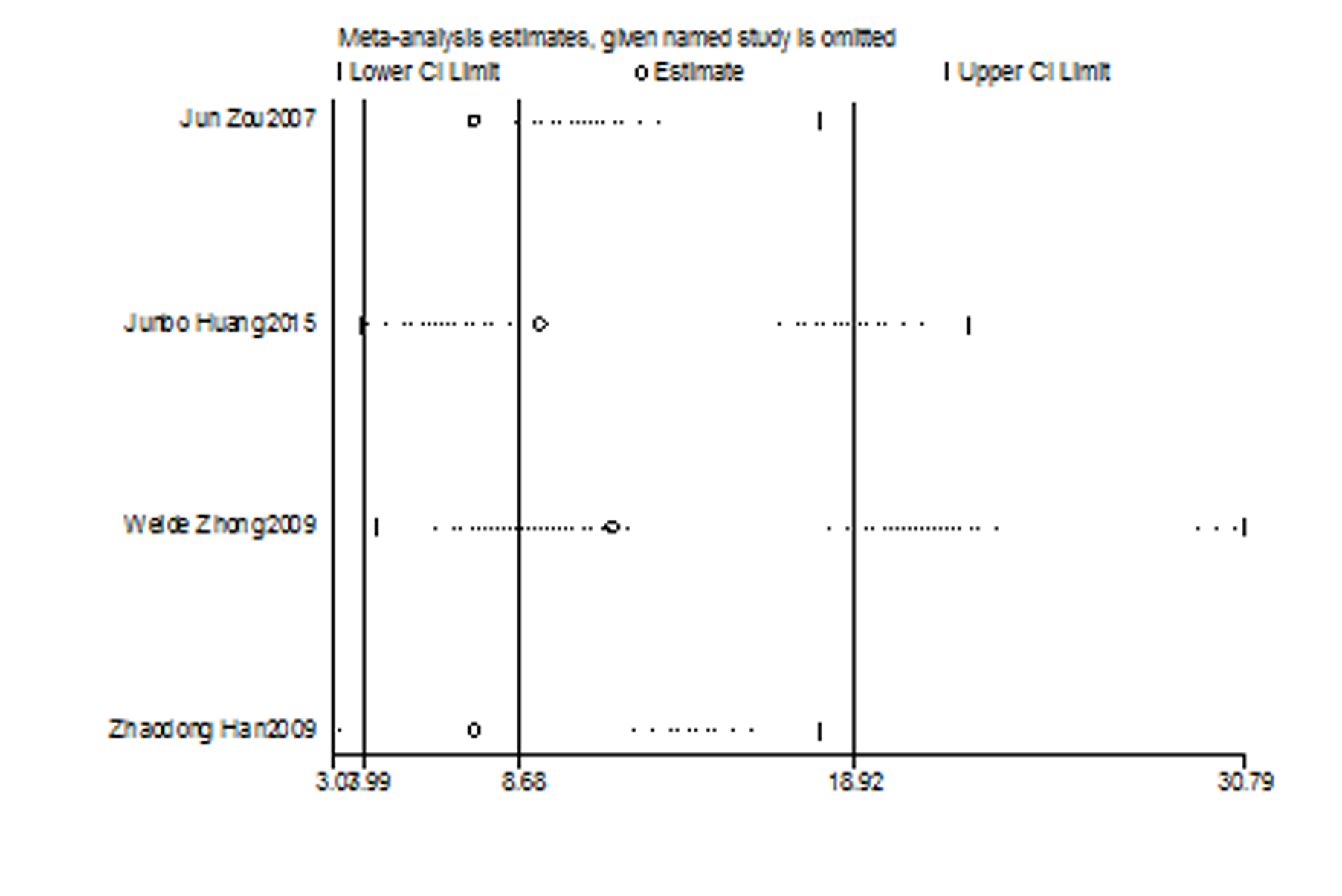

Supplement: S5 Fig — (TIF) [file pone.0163678.s005.tif]

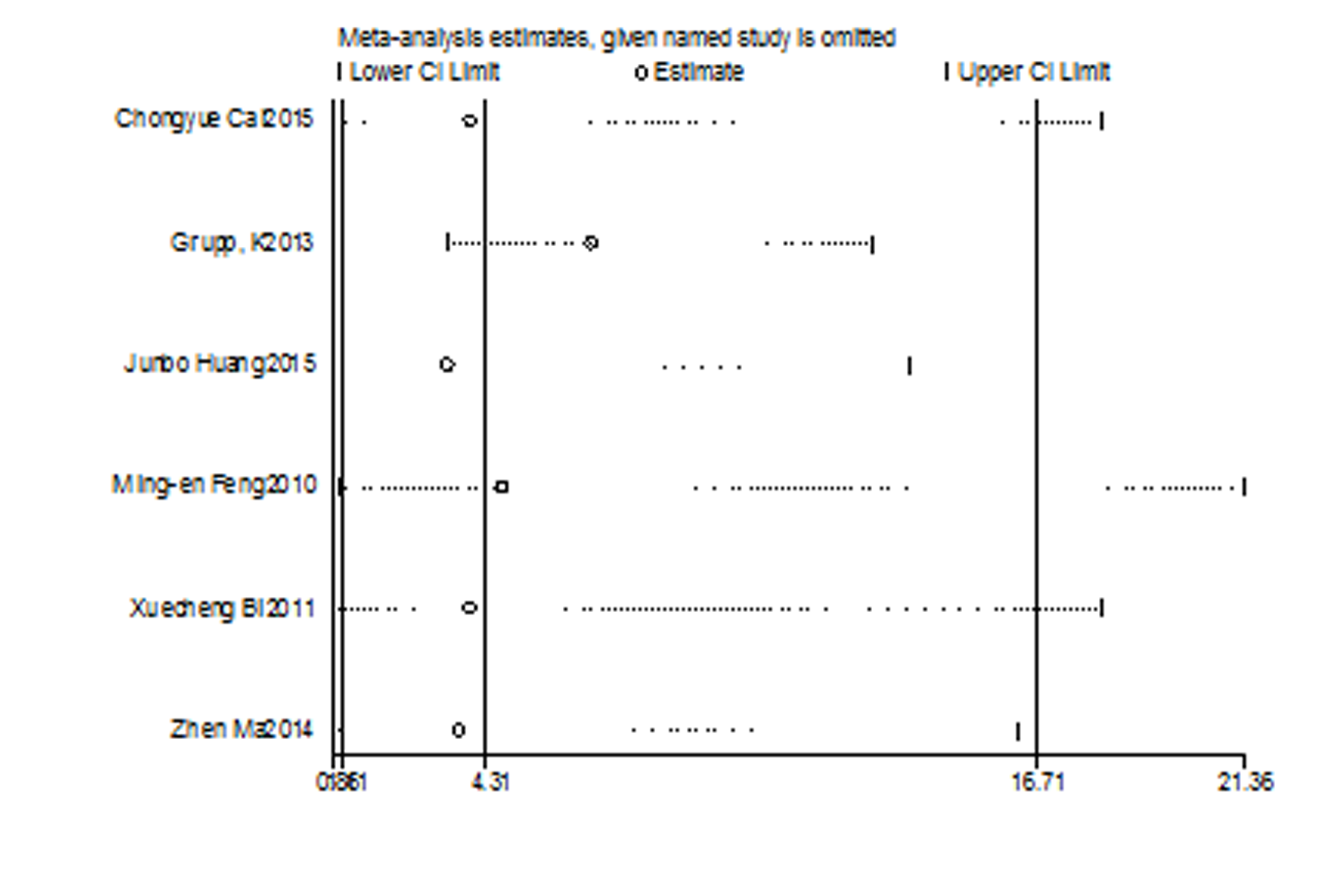

Supplement: S6 Fig — (TIF) [file pone.0163678.s006.tif]

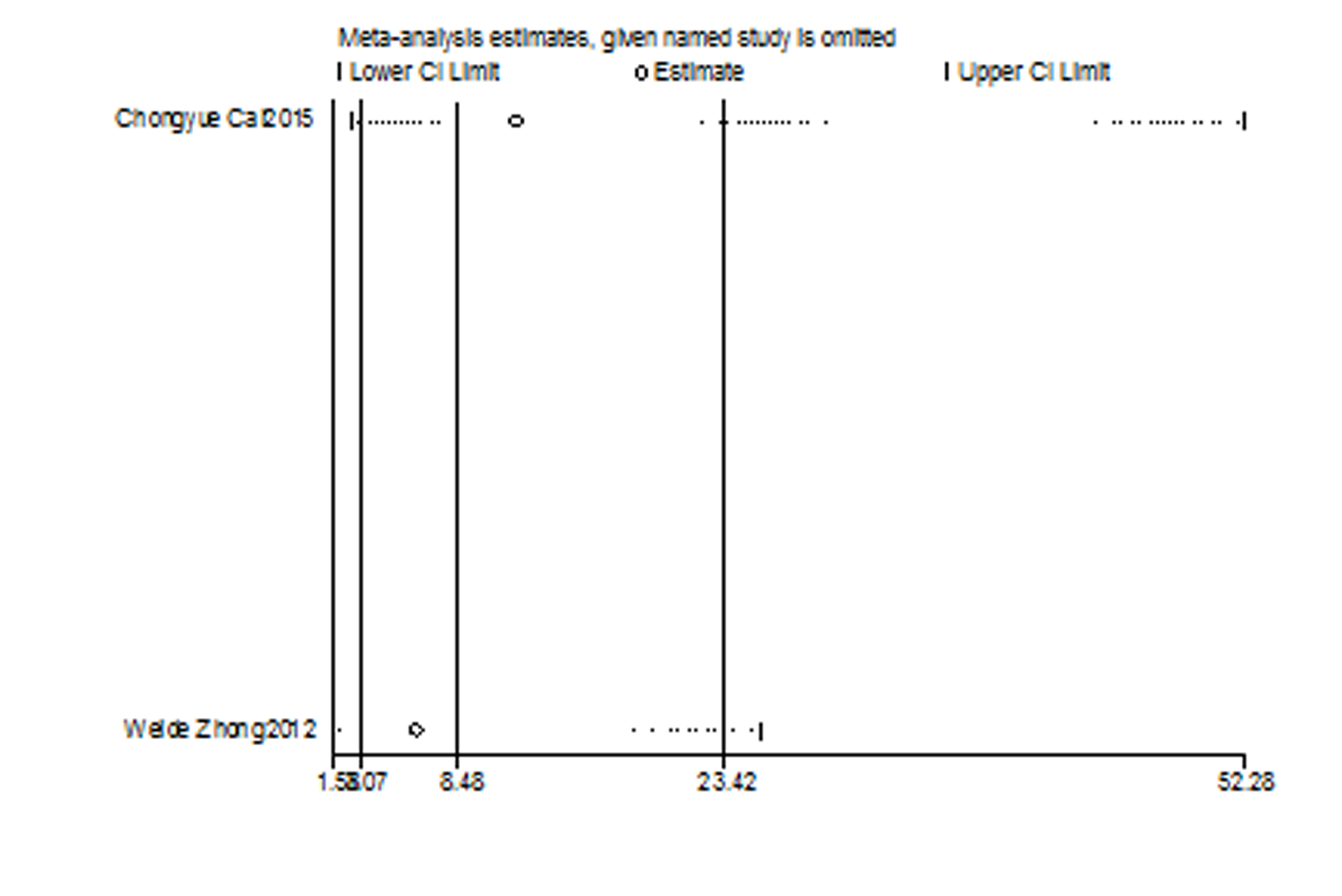

Supplement: S7 Fig — (TIF) [file pone.0163678.s007.tif]
