# Supplementary material for: CD147 and Prostate Cancer: A Systematic Review and Meta-Analysis
Source: PLoS One. 2016 Sep 29;11(9):e0163678. doi: 10.1371/journal.pone.0163678 (PMC5042541; doi:10.1371/journal.pone.0163678)
Supplement: S1 File — (DOCX) [file pone.0163678.s008.docx]

**Search strategy**

**(A)Pubmed**

**41 articles**

**Prostate**

**[MeSH Terms] OR Prostatic Diseases[MeSH Terms] OR Prostat*[Title/Abstract] OR prostatic[Title/Abstract] OR Prostatomegaly[Title/Abstract] OR prostate[Title/Abstract]**

**CD147**

**antigens, cd147[MeSH Terms] OR cd147[Title/Abstract] OR (extracellular[Title/Abstract] AND matrix[Title/Abstract] AND metalloproteinase[Title/Abstract] AND inducer[Title/Abstract]) OR "extracellular matrix metalloproteinase inducer"[Title/Abstract] OR EMMPRIN[Title/Abstract]**

**Total search**

**(prostate[MeSH Terms] OR Prostatic Diseases[MeSH Terms] OR Prostat*[Title/Abstract] OR prostatic[Title/Abstract] OR Prostatomegaly[Title/Abstract] OR prostate[Title/Abstract]) AND (antigens, cd147[MeSH Terms] OR cd147[Title/Abstract] OR (extracellular[Title/Abstract] AND matrix[Title/Abstract] AND metalloproteinase[Title/Abstract] AND inducer[Title/Abstract]) OR "extracellular matrix metalloproteinase inducer"[Title/Abstract] OR EMMPRIN[Title/Abstract])**

**(B)Embase**

**72 articles**

**Prostate**

**'prostate'/exp OR 'prostate disease'/exp OR Prostat*:ti,ab OR prostatic:ti,ab OR Prostatomegaly:ti,ab OR prostate:ti,ab**

**CD147**

**'CD147 antigen'/exp OR cd147:ti,ab OR (extracellular:ti,ab AND matrix:ti,ab AND metalloproteinase:ti,ab AND inducer:ti,ab) OR "extracellular matrix metalloproteinase inducer":ti,ab OR EMMPRIN:ti,ab**

**Total search**

**('prostate'/exp OR 'prostate disease'/exp OR Prostat*:ti,ab OR prostatic:ti,ab OR Prostatomegaly:ti,ab OR prostate:ti,ab) AND ('CD147 antigen'/exp OR cd147:ti,ab OR (extracellular:ti,ab AND matrix:ti,ab AND metalloproteinase:ti,ab AND inducer:ti,ab) OR "extracellular matrix metalloproteinase inducer":ti,ab OR EMMPRIN:ti,ab) AND [embase]/lim**

**(C)Cochrane Library**

**1 article**

**Prostate**

**[mh prostate] OR [mh "Prostatic Diseases"] OR Prostat*:ti,ab OR prostatic:ti,ab OR Prostatomegaly:ti,ab OR prostate:ti,ab**

**CD147**

**[mh "Antigens, CD147"] OR cd147:ti,ab OR (extracellular:ti,ab AND matrix:ti,ab AND metalloproteinase:ti,ab AND inducer:ti,ab) OR "extracellular matrix metalloproteinase inducer":ti,ab OR EMMPRIN:ti,ab**

**Total search**

**([mh prostate] OR [mh "Prostatic Diseases"] OR Prostat*:ti,ab OR prostatic:ti,ab OR Prostatomegaly:ti,ab OR prostate:ti,ab) AND ([mh "Antigens, CD147"] OR cd147:ti,ab OR (extracellular:ti,ab AND matrix:ti,ab AND metalloproteinase:ti,ab AND inducer:ti,ab) OR "extracellular matrix metalloproteinase inducer":ti,ab OR EMMPRIN:ti,ab)**

**(D)WOS**

**70 articles**

**Prostate**

**TS=prostate OR TS=(Prostatic AND Diseases) OR TS=Prostat* OR TS=prostatic OR TS=Prostatomegaly OR TS=prostate**

**CD147**

**TS=Antigens, CD147 OR TS=cd147 OR (TS=extracellular AND TS=matrix AND TS=metalloproteinase AND TS=inducer) OR TS="extracellular matrix metalloproteinase inducer" OR TS=EMMPRIN**

**Total search**

**(TS=prostate OR TS=(Prostatic AND Diseases) OR TS=Prostat* OR TS=prostatic OR TS=Prostatomegaly OR TS=prostate) AND (TS=Antigens, CD147 OR TS=cd147 OR (TS=extracellular AND TS=matrix AND TS=metalloproteinase AND TS=inducer) OR TS="extracellular matrix metalloproteinase inducer" OR TS=EMMPRIN)**

**(E)CNKI**

**38 articles**

**Prostate**

**SU='前列腺' OR SU='前列腺癌' OR SU='前列腺肿瘤' OR SU='前列腺增生' OR SU='前列腺疾病'**

**CD147**

**SU='CD147' OR SU='抗原, CD147' OR SU='细胞外基质金属蛋白酶诱导因子'**

**Total search**

**(SU='前列腺' OR SU='前列腺癌' OR SU='前列腺肿瘤' OR SU='前列腺增生' OR SU='前列腺疾病') AND (SU='CD147' OR SU='抗原, CD147' OR SU='细胞外基质金属蛋白酶诱导因子')**

**(F)WANFANG**

**37 articles**

**Prostate**

**主题:(前列腺) + 主题:(前列腺癌) + 主题:(前列腺肿瘤) + 主题:(前列腺增生) + 主题:(前列腺疾病)**

**CD147**

**主题:(CD147) + 主题:(抗原, CD147) + 主题:(细胞外基质金属蛋白酶诱导因子)**

**Total search**

**(主题:(前列腺) + 主题:(前列腺癌) + 主题:(前列腺肿瘤) + 主题:(前列腺增生) + 主题:(前列腺疾病)) * (主题:(CD147) + 主题:(抗原, CD147) + 主题:(细胞外基质金属蛋白酶诱导因子)**
