# Supplementary material for: CD147 and Prostate Cancer: A Systematic Review and Meta-Analysis
Source: PLoS One. 2016 Sep 29;11(9):e0163678. doi: 10.1371/journal.pone.0163678 (PMC5042541; doi:10.1371/journal.pone.0163678)
Supplement: S2 File — (DOC) [file pone.0163678.s009.doc]

| **Section/topic** | **#** | **Checklist item** | **Reported on page #** |
| --- | --- | --- | --- |
| **TITLE** | | |  |
| Title | 1 | A Systematic Review and Meta-Analysis | 7 |
| **ABSTRACT** | | |  |
| Structured summary | 2 | Background: Prostate cancer is one of the most common non-cutaneous malignancies in men. We aimed to systemically evaluate the relationship between the expression of CD147 in tissues and the clinicopathological features of prostate cancer.  Methods and Findings: PubMed (1966–2016), EMBASE (1980–2016), the Cochrane Library (1996–2016), Web of Science (1945–2016), China National Knowledge Infrastructure (1982–2016), and the WanFang databases (1988–2016) were searched. Literature quality assessment was performed with the Newcastle-Ottawa Scale. Meta-analysis was performed by using Review Manager 5.3 and Stata 13.0. A total of 12591 prostate cancer patients from 14 studies were included. The results of the meta-analysis showed that there were significant differences in the positive expression rate in the following comparisons: prostatic cancer tissues vs. normal prostate tissues (odds ratio [OR] = 26.93, 95% confidence interval [CI] 7.95–91.20, P < 0.00001), prostatic cancer tissues vs. benign prostatic hyperplasia tissues (OR = 20.54, 95% CI 8.20–51.44, P < 0.00001), high Gleason score vs. low Gleason score (OR = 2.39, 95% CI 1.33–4.27, P = 0.03), TNM III to IV vs. TNM I to II (OR = 9.95, 95% CI 4.96–19.96, P < 0.00001), low or moderate differentiation vs. high differentiation (OR = 8.12, 95% CI 3.69–17.85, P < 0.00001), lymph node metastasis vs. non-lymph node metastasis (OR = 4.31, 95% CI 1.11–16.71, P = 0.03), and distant metastasis vs. non-distant metastasis (OR = 8.90, 95% CI 3.24–24.42, P < 0.00001).  Conclusion: The CD147 positive expression rate was closely related to the clinical characteristics of prostate cancer, but more research is needed to confirm the findings owing to the results of the subgroups. | 8-9 |
| **INTRODUCTION** | | |  |
| Rationale | 3 |  |  |
| Objectives | 4 | to systemically evaluate the relationship between the expression of CD147 in tissues and the clinicopathological features of prostate cancer | 10-11 |
| **METHODS** | | |  |
| Protocol and registration | 5 |  |  |
| Eligibility criteria | 6 | 1. Published case control study or randomized controlled trial that provides original data about CD147 and prostate cancer with clinical pathological characteristics; 2. All cases had complete clinical and pathological data, without radiotherapy or chemotherapy before sampling; 3. Pathological sections were all studied, and CD147 was detected by immunohistochemical staining; 4. When there was duplicate publication or similar information, the best quality study was retained. | 11 |
| Information sources | 7 | PubMed (1966–2016), EMBASE (1980–2016), the Cochrane Library (1996–2016), Web of Science (1945–2016), China National Knowledge Infrastructure (1982–2016), and the WanFang databases (1988–2016) were searched | 12 |
| Search | 8 | The following combined search term was used: (prostate, Prostatic, Prostat*, Prostatomegaly) AND (CD147, extracellular matrix metalloproteinase inducer, EMMPRIN). We combined the term appropriately with MeSH Terms and used an appropriate adjustment for different databases. Details of the search strategies can be found in Appendix 1 | 12 |
| Study selection | 9 | A total of 259 studies were identified, and 95 studies were excluded because of duplication. After reading the titles and abstracts, 114 studies were excluded. Fifty full text studies were carefully reviewed (excluded for being animal studies [n = 19]; reviews and meta-analyses [n = 1]; reported CD147 mRNA expression [n = 1]; no control group [n = 2]; and completely irrelevant [n = 12]**).** Finally, 15 trials were included for qualitative analysis and 14 trials were included for quantitative analysis | 14 |
| Data collection process | 10 | the full text articles were independently reviewed by two authors to see if they met the inclusion criteria, and differences of opinion were resolved by a third author. All of the data were extracted independently by two authors. The corresponding author of each study was contacted to provide information on missing or incomplete data. | 12 |
| Data items | 11 |  |  |
| Risk of bias in individual studies | 12 | The Newcastle-Ottawa quality assessment scale of case control studies (NOS) [16] was adopted to assess the quality of included studies. It has three categories (selection, comparability, and exposure) and eight items. Two researchers performed the quality assessments separately. In the selection category (adequate definition of the cases, representativeness of the cases, selection of controls, definition of controls) and exposure category (ascertainment of exposure, same method of ascertainment for cases and controls, non-response rate), a quality research item received one star, and a comparable category (comparability of cases and controls on the basis of the design or analysis) could receive at most two stars. The quality assessment values ranged from 0 to 9 stars. Each band indicates the percentage of the included studies  that met each of these 109 quality criteria. | 14 |
| Summary measures | 13 | Results were expressed as odds ratios (OR) and 95% confidence intervals (95% CI) | 13 |
| Synthesis of results | 14 | A fixed-effects model was adopted in the case of no evidence of significant heterogeneity (P > 0.1 and I2 < 50%); otherwise, a random-effects model was used. If possible, heterogeneity was explored and subgroup analyses were performed. Subgroup analyses were conducted based on patient age and study area. If heterogeneity could not be explored, we conducted a sensitivity analysis to identify the study with the most heterogeneity. | 13 |

Page 1 of 2

| **Section/topic** | **#** | **Checklist item** | **Reported on page #** |
| --- | --- | --- | --- |
| Risk of bias across studies | 15 | If publication bias was confirmed, a trim-and-fill method developed by Duval and Tweedie was implemented to adjust for this bias.Then, we replicated the funnel plot with their ‘‘missing’’counterparts around the adjusted 132 summary estimate | 13 |
| Additional analyses | 16 | Sensitive analysis was also performed to evaluate the influences of individual studies on the final effect size. When some studies were omitted or subgroup analyses were performed, if no decreases in heterogeneity were observed, a qualitative systematic review method was used to describe the results. All P values were 2-sided, and P < 0.05 was considered significant. Egger’s test was used to assess publication bias (P < 0.05 was considered statistically significant) | 13 |
| **RESULTS** | | |  |
| Study selection | 17 | A total of 259 studies were identified, and 95 studies were excluded because of duplication. After reading the titles and abstracts, 114 studies were excluded. Fifty full text studies were carefully reviewed (excluded for being animal studies [n = 19]; reviews and meta-analyses [n = 1]; reported CD147 mRNA expression [n = 1]; no control group [n = 2]; and completely irrelevant [n = 12]**).** Finally, 15 trials were included for qualitative analysis and 14 trials were included for quantitative analysis | 14 |
| Study characteristics | 18 | A total of 15 studies [9,10,11,12,13,14,15,17,18,19,20,21,22,23,40,] were identified for qualitative analysis. The study by Bauman et al. was not included in the quantitative analysis because it provided the mean optical density of CD147 and not the positive expression rate of CD147. Therefore, 14 studies [9,10,11,12,13,14,17,18,19,20,21,22,23,40,] were included for quantitative analysis, including 12,591 prostate cancer tissues, 298 benign prostatic hyperplasia tissues, and 175 normal prostate tissues. Six articles provided a positivity rate of CD147 expression in prostate cancer and normal prostate tissues, 8 articles reported on CD147 expression in prostate cancer and benign prostatic hyperplasia, 12 articles involved Gleason score, 6 articles involved TNM stage, 5 articles involved differentiation, 6 articles involved lymph node metastasis, and 2 articles involved distant metastasis.  Eleven of the studies were performed in Asia and 3 elsewhere. All 14 studies adopted immunohistochemistry (IHC) as the detection method, but the method for judging negative and positive staining was different among studies. For example, Wang used an IHC score. However, Nelma Pertega-Gomes, Grupp K, and Weide Zhong used cell staining intensity (Nelma Pertega-Gomes and Grupp K’s cut-off value was 0%, while Weide Zhong’s was 5%). The source of antibodies in all included studies was primarily three places (Fourth Military Medical University, Sigma, and ZYMED). The tissue slice thickness across the studies had some minor differences. | 14 |
| Risk of bias within studies | 19 | All 14 studies adopted immunohistochemistry (IHC) as the detection method, but the method for judging negative and positive staining was different among studies. For example, Wang used an IHC score. However, Nelma Pertega-Gomes, Grupp K, and Weide Zhong used cell staining intensity (Nelma Pertega-Gomes and Grupp K’s cut-off value was 0%, while Weide Zhong’s was 5%). The source of antibodies in all included studies was primarily three places (Fourth Military Medical University, Sigma, and ZYMED). The tissue slice thickness across the studies had some minor differences. | 14-15 |
| Results of individual studies | 20 |  |  |
| Synthesis of results | 21 | The results of the meta-analysis showed that there were significant differences in the following comparisons: prostatic cancer tissues vs. normal prostate tissues (odds ratio [OR] = 26.93, 95% confidence interval [CI] 7.95–91.20, P < 0.00001), prostatic cancer tissues vs. benign prostatic hyperplasia tissues (OR = 20.54, 95% CI 8.20–51.44, P < 0.00001), high Gleason score vs. low Gleason score (OR = 2.39, 95% CI 1.33–4.27, P = 0.03), TNM III to IV vs. TNM I to II (OR = 9.95, 95% CI 4.96–19.96, P < 0.00001), low or moderate differentiation vs. high differentiation (OR = 8.12, 95% CI 3.69 –17.85 P < 0.00001), lymph node metastasis vs. non-lymph node metastasis (OR = 4.31, 95% CI 1.11–16.71, P = 0.03), and distant metastasis vs. non-distant metastasis (OR = 8.90, 95% CI 3.24–24.42, P < 0.00001). | 15-19 |
| Risk of bias across studies | 22 | Egger’s test (as shown in Table 3) and funnel plots were used to evaluate the publication bias of these studies. Publication bias was found in the Gleason score (P = 0.003) and TNM stage (P = 0.038) but the other outcomes suggested no evidence of publication bias. We used a trim-and-fill method to adjust for the publication bias. For the Gleason score, we first excluded four studies which had a smaller number of patients. The adjusted summary showed there was no significant difference between high Gleason score and low Gleason score (OR = −0.083, 95%CI −0.197 to 0.032, P = 0.156) and there was no heterogeneity. Then, we added four studies that showed the opposite conclusion. The adjusted summary suggested there was a significant difference between cancers with a high Gleason score and a low Gleason score (OR = −0.237, 95% CI −0.346 to −0.129, P <0.0001) without heterogeneity. However, our conclusion about the Gleason score should be interpreted with caution and it needs more research to support it. The adjusted results of the TNM stage, after one article was excluded and nothing was added, suggested a similar conclusion, that there was a significant difference between TNM III and IV and TNM I and II. This was consistent with our prior conclusion and indicates our results are statistically robust | 19 |
| Additional analysis | 23 | Influence analyses, in which one study was removed at a time, were performed for each meta-analysis to evaluate the stability of the results. These analyses showed the corresponding OR was not significantly altered and suggests our results are stable | 19 |
| **DISCUSSION** | | |  |
| Summary of evidence | 24 |  |  |
| Limitations | 25 | There are some limitations to our study. First of all, 12,591 prostate cancers were included in the 14 studies, but not every outcome had that 336 many samples. For some outcomes (such as lymph node metastasis) there were a relatively small number of samples, and they need more trials to confirm our conclusions. Second, although we tried to collect all relevant date from these studies, some data could still be missing, which means that publication bias may be unavoidable. Third, the cut-off value was different among some studies, which had inconsistent definitions for “negative” and“positive,” leading to between-study heterogeneity. Therefore, the results need to be interpreted with caution. In addition, some unpublished trials that had negative outcomes were not included. | 22-23 |
| Conclusions | 26 | The results of this review suggest that CD147 positivity is higher in prostate cancer than in benign prostatic hyperplasia and normal prostate tissue. Furthermore, the positivity rate of CD147 expression is associated with Gleason score, TNM stage, differentiation, lymph node metastasis, and distant metastasis. However, the conclusion that CD147 positivity rate was higher in prostatic cancer tissues vs. benign prostatic hyperplasia tissues, high Gleason score vs. low Gleason score, and lymph node metastasis vs. non-lymph node metastasis should be interpreted with caution and needs to be confirmed owing to the different results in the subgroup analyses. | 23 |
| **FUNDING** | | |  |
| Funding | 27 |  |  |

*From:*  Moher D, Liberati A, Tetzlaff J, Altman DG, The PRISMA Group (2009). Preferred Reporting Items for Systematic Reviews and Meta-Analyses: The PRISMA Statement. PLoS Med 6(7): e1000097. doi:10.1371/journal.pmed1000097

For more information, visit: **www.prisma-statement.org**.

Page 2 of 2
