# Supplementary material for: CD147 and Prostate Cancer: A Systematic Review and Meta-Analysis
Source: PLoS One. 2016 Sep 29;11(9):e0163678. doi: 10.1371/journal.pone.0163678 (PMC5042541; doi:10.1371/journal.pone.0163678)
Supplement: S1 Table — (DOCX) [file pone.0163678.s010.docx]

Table S1.The results of subgroup analysis

| Stratified analysis | No. of studies | Odds ratio | | Model | Heterogeneity | |
| --- | --- | --- | --- | --- | --- | --- |
|  |  | OR (95% CI) | P value |  | I2 (%) | P value |
| ① |  |  |  |  |  |  |
| Country |  |  |  |  |  |  |
| Asian | 5 | 39.19[17.83,86.16] | <0.00001 | Fixed | 47 | 0.11 |
| Non-Asian | 1 | 5.57[[1.89,16.40] | 0.002 |  |  |  |
| Antibody source |  |  |  |  |  |  |
| Biological Research Center  of Fourth Military Medical University | 3 | 27.27[9.56,77.81] | <0.00001 | Fixed | 0 | 0.84 |
| Sigma | 1 | 1435.91[77.59,26574.47] | <0.00001 |  |  |  |
| ZYMED | 1 | 5.57[1.89,16.40] | 0.002 |  |  |  |
| Other antibodies | 1 | 18.00[3.81,85.06] | 0.0003 |  |  |  |
| Scoring system |  |  |  |  |  |  |
| IHC score | 3 | 66.92[8.76,511.43] | <0.00001 | Random | 72 | 0.03 |
| Ratios of stained cell | 3 | 11.80[5.07,27.42] | <0.00001 | Fixed | 30 | 0.24 |
| ② |  |  |  |  |  |  |
| Country |  |  |  |  |  |  |
| Asian | 8 | 20.54[8.20,51.44] | <0.00001 | Random | 71 | 0.0009 |
| Non-Asian | 1 | 12.07[2.60,56.04] | 0.001 |  |  |  |
| Antibody source |  |  |  |  |  |  |
| Biological Research Center  of Fourth Military Medical University | 3 | 15.79[5.00,49.89] | <0.00001 | Random | 68 | 0.04 |
| Sigma | 1 | 644.76[36.27,11461.40] | <0.00001 |  |  |  |
| ZYMED | 2 | 13.20[5.15,33.83] | <0.00001 | Fixed | 0 | 0.88 |
| Other antibodies | 2 | 39.56[0.27,5824.35] | 0.15 | Random | 90 | 0.002 |
| Scoring system |  |  |  |  |  |  |
| IHC score | 5 | 47.38[9.62,233.47] | <0.00001 | Random | 82 | 0.0002 |
| Ratios of stained cell | 3 | 9.20[4.39,19,30] | <0.00001 | Fixed | 0 | 0.38 |
| ③ |  |  |  |  |  |  |
| Country |  |  |  |  |  |  |
| Asian | 7 | 3.57[2.52,5.06] | <0.00001 | Fixed | 0 | 0.57 |
| Non-Asian | 3 | 1.66[0.63,4.35] | 0.3 | Random | 88 | 0.0003 |
| Antibody source |  |  |  |  |  |  |
| Biological Research Center  of Fourth Military Medical University | 3 | 3.01[2.02,4.48] | <0.00001 | Fixed | 0 | 0.43 |
| Sigma | 1 | 16.91[0.92,312.40] | 0.06 |  |  |  |
| ZYMED | 3 | 2.91[1.76,4.81] | <0.00001 | Fixed | 11 | 0.32 |
| Other antibodies | 3 | 2.35[0.52,10.71] | 0.27 | Random | 83 | 0.003 |
| Scoring system |  |  |  |  |  |  |
| IHC score | 5 | 1.59[0.93,2.73] | 0.09 | Fixed | 49 | 0.1 |
| Ratios of stained cell | 7 | 2.70[1.26,5.79] | 0.01 | Random | 91 | <0.00001 |
| ④ |  |  |  |  |  |  |
| Country |  |  |  |  |  |  |
| Asian | 6 | 9.95[4.96,19.96] | <0.00001 | Fixed | 0 | 0.97 |
| Non-Asian | 0 |  |  |  |  |  |
| Antibody source |  |  |  |  |  |  |
| Biological Research Center  of Fourth Military Medical University | 3 | 8.11[2.79,23.54] | 0.0001 | Fixed | 0 | 0.92 |
| Sigma | 2 | 12.28[5.20,29.03] | <0.00001 | Fixed | 0 | 0.9 |
| ZYMED | 0 |  |  |  |  |  |
| Other antibodies | 1 |  |  |  |  |  |
| Scoring system |  |  |  |  |  |  |
| IHC score | 0 |  |  |  |  |  |
| Ratios of stained cell | 6 | 9.95[4.96,19.96] | <0.00001 | Fixed | 0 | 0.97 |
| ⑤ |  |  |  |  |  |  |
| Country |  |  |  |  |  |  |
| Asian | 4 | 8.12[3.69,17.85] | <0.00001 | Fixed | 0 | 0.65 |
| Non-Asian | 0 |  |  |  |  |  |
| Antibody source |  |  |  |  |  |  |
| Biological Research Center  of Fourth Military Medical University | 3 | 8.75[3.63,21.09] | <0.00001 | Fixed | 0 | 0.48 |
| Sigma | 1 | 6.25[1.08,36.08] | 0.04 |  |  |  |
| ZYMED | 0 |  |  |  |  |  |
| Other antibodies | 0 |  |  |  |  |  |
| Scoring system |  |  |  |  |  |  |
| IHC score | 4 | 8.12[3.69,17.85] | <0.00001 | Fixed | 0 | 0.65 |
| Ratios of stained cell | 0 |  |  |  |  |  |
| ⑥ |  |  |  |  |  |  |
| Country |  |  |  |  |  |  |
| Asian | 5 | 6.84[3.51,13.32] | <0.00001 | Fixed | 0 | 0.82 |
| Non-Asian | 1 | 0.74[0.59,0.92] | 0.006 |  |  |  |
| Antibody source |  |  |  |  |  |  |
| Biological Research Center  of Fourth Military Medical University | 1 | 6.28[1.86,21.22] | 0.003 |  |  |  |
| Sigma | 1 | 25.54[1.38,473.42] | 0.03 |  |  |  |
| ZYMED | 1 | 8.08[2.24,29.17] | 0.001 |  |  |  |
| Other antibodies | 3 | 2.09[0.40,10.92] | 0.38 | Random | 85 | 0.002 |
| Scoring system |  |  |  |  |  |  |
| IHC score | 2 | 8.04[2.65,24.41] | 0.0002 | Fixed | 0 | 0.38 |
| Ratios of stained cell | 4 | 2.98[0.63,14.07] | 0.17 | Random | 88 | <0.00001 |
| ⑦ |  |  |  |  |  |  |
| Country |  |  |  |  |  |  |
| Asian | 2 | 8.90[3.24,24.42] | <0.00001 | Fixed | 0 | 0.54 |
| Non-Asian | 0 |  |  |  |  |  |
| Antibody source |  |  |  |  |  |  |
| Biological Research Center  of Fourth Military Medical University | 1 | 11.84[2.68,52.28] | 0.001 |  |  |  |
| Sigma | 0 |  |  |  |  |  |
| ZYMED | 0 |  |  |  |  |  |
| Other antibodies | 1 | 6,33[1.58,25.45] | 0.009 |  |  |  |
| Scoring system |  |  |  |  |  |  |
| IHC score | 0 |  |  |  |  |  |
| Ratios of stained cell | 2 | 8.90[3.24,24.42] | <0.00001 | Fixed | 0 | 0.54 |

Note: ①. CD147 with prostatic cancer tissues and normal prostate tissues; ②. CD147 with prostatic cancer tissues and benign prostatic hyperplasia tissues; ③. CD147 with Gleason score of prostatic cancer tissues; ④. CD147 with TNM stage of prostatic cancer tissues; ⑤. CD147 with differentiation of prostatic cancer tissues; ⑥. CD147 with lymph node metastasis of prostatic cancer tissues; ⑦. CD147 with distant metastasis of prostatic cancer tissues.
